# Effects of experimental hypovolemia and pain on pre‐ejection period and pulse transit time in healthy volunteers

**DOI:** 10.14814/phy2.15355

**Published:** 2022-06-24

**Authors:** Håvard Djupedal, Torkjell Nøstdahl, Jonny Hisdal, Svein Aslak Landsverk, Lars Øivind Høiseth

**Affiliations:** ^1^ Department of Anesthesiology Telemark Hospital Skien Norway; ^2^ University of Oslo Oslo Norway; ^3^ Department of Vascular Surgery Division of Cardiovascular and Pulmonary Diseases, Oslo University Hospital Oslo Norway; ^4^ Department of Anesthesiology and Intensive Care Oslo University Hospital Oslo Norway; ^5^ Norwegian Air Ambulance Foundation Oslo Norway

**Keywords:** hypovolemia, non‐invasive, pre‐ejection period, pulse transit time

## Abstract

Trauma patients may suffer significant blood loss, and noninvasive methods to diagnose hypovolemia in these patients are needed. Physiologic effects of hypovolemia, aiming to maintain blood pressure, are largely mediated by increased sympathetic nervous activity. Trauma patients may however experience pain, which also increases sympathetic nervous activity, potentially confounding measures of hypovolemia. Elucidating the common and separate effects of the two stimuli on diagnostic methods is therefore important. Lower body negative pressure (LBNP) and cold pressor test (CPT) are experimental models of central hypovolemia and pain, respectively. In the present analysis, we explored the effects of LBNP and CPT on pre‐ejection period and pulse transit time, aiming to further elucidate the potential use of these variables in diagnosing hypovolemia in trauma patients. We exposed healthy volunteers to four experimental sequences with hypovolemia (LBNP 60 mmHg) or normovolemia (LBNP 0 mmHg) and pain (CPT) or no pain (sham) in a 2 × 2 fashion. We calculated pre‐ejection period and pulse transit time from ECG and ascending aortic blood velocity (suprasternal Doppler) and continuous noninvasive arterial pressure waveform (volume‐clamp method). Fourteen subjects were available for the current analyses. This experimental study found that pre‐ejection period increased with hypovolemia and remained unaltered with pain. Pulse transit time was reduced by pain and increased with hypovolemia. Thus, the direction of change in pulse transit time has the potential to distinguish hypovolemia and pain.

## Graphical abstract

In this paper, we found that pulse transit time changed in opposite directions when healthy volunteers were exposed to experimental hypovolemia and pain, respectively. Interestingly, the direction of change in pulse transit time can potentially be used to distinguish hypovolemia and pain, two stimuli that both activate the sympathetic nervous system.

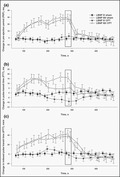

## 1 INTRODUCTION

Hypovolemia from blood loss contributes substantially to morbidity and mortality in trauma patients (Eastridge et al., 2019), but may be difficult to diagnose and quantify. Advanced invasive monitoring is usually not available in out‐of‐hospital setting or in the initial phase in the emergency room, and clinicians must therefore rely on changes in traditional vital signs such as heart rate (HR), blood pressure (BP), pulse pressure (PP), and respiratory rate (RR) for assessment of hypovolemia (Convertino et al., 2006; Zhu et al., 2019). The physiologic responses to hypovolemia are largely mediated by increased sympathetic nervous activity (Schadt & Ludbrook, 1991; Yadav et al., 2017). Other sympathetic stimuli occurring in trauma patients, such as pain, may thereby confound diagnostic methods in these patients (Brotman et al., 2007; Guly et al., 2011). It is therefore important to elucidate the effects of both hypovolemia and pain on diagnostic methods of hypovolemia intended for use in trauma patients (Liu et al., 2017). Systolic time intervals and vascular transit times reflect changes in cardiac function and volume status and have been proposed as measures of hypovolemia (Chan et al., 2007; Newlin & Levenson, 1979).

Lower body negative pressure (LBNP) is an experimental model where the integrated compensatory responses to hypovolemia can be studied in healthy volunteers. Negative pressure is applied to the lower part of the body, causing pooling of blood in the lower body and a central (upper body) hypovolemia (Goswami et al., 2019). Hypovolemia and LBNP activate cardiovascular reflexes, mediated by the sympathetic nervous system, to maintain mean arterial pressure (MAP) by increasing HR, cardiac contractility, and arterial and venous vascular tone. After an initial parasympathetic tone reduction, increasing LBNP leads to a linear increase in efferent sympathetic traffic to the heart and peripheral vasculature (Cooke et al., 2009; Goswami et al., 2019).

Cold pressor test (CPT) is a model of acute pain, where typically a hand is immersed in ice‐cold water (Wolf & Hardy, 1941). CPT stimulates thermoreceptors and nociceptors (Patapoutian et al., 2003), causing increased sympathetic nervous activity (Victor et al., 1987) and a rise in arterial pressure, peripheral resistance, HR, and cardiac output (Stens et al., 2020; Stocks et al., 2004).

We have previously reported on the effects of LBNP and CPT on central and peripheral perfusion in healthy volunteers (Høiseth et al., 2015). In the present analysis, using data from the same study, we explored the effects of these stimuli on changes in pre‐ejection period and pulse transit time. Pre‐ejection period was defined as the time from the ECG R‐wave to ejection of blood into the ascending aorta after aortic valve opening. Pulse transit time was defined as the time from the ECG R‐wave to the following peripheral pulse (Loukogeorgakis et al., 2002), which has been the most common way to define pulse transit time the last four decades (Ding & Zhang, 2019). The aim of the present study in healthy volunteers was to explore the effects of experimental hypovolemia and pain in isolation and combination on pre‐ejection period and pulse transit time. The purpose was to model how pre‐ejection period and pulse transit time would perform in diagnosing hypovolemia in patients experiencing pain, simulating for example, trauma patients.

## 2 MATERIALS AND METHODS

The data for the present analysis were sampled for a study which has previously been published (Høiseth et al., 2015), approved by the regional ethics committee (REK sør‐øst C, 2012/790). Twenty healthy volunteers were included after written informed consent.

### 2.1 Experimental protocol

**FIGURE 1:**
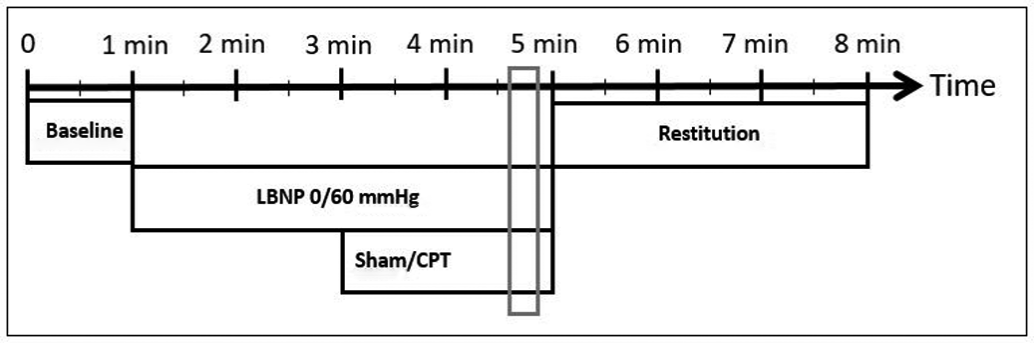
Experimental sequences. The gray bar indicates the point where we have done our analyses, which represent the last 30 s of interventions. Lower body negative pressure (LBNP). Cold pressor test (CPT).

While placed in the LBNP‐chamber (Hisdal et al., 2003), the subjects were unaware of the order and the number of CPT interventions. The subjects were exposed to either normovolemia with no negative pressure in the LBNP‐chamber (LBNP 0) or hypovolemia with a chamber pressure of −60 mmHg (LBNP 60). We placed a bucket that was either empty (sham) or filled with a mixture of ice and water (CPT), so that the right hand could be immersed to the wrist. We exposed the subjects to four experimental sequences with either LBNP 0 or LBNP 60 and no pain (sham) or pain (CPT) in a 2 × 2 fashion, each lasting 8 min (Figure 1). In the first and third sequences, we applied no negative pressure in the LBNP chamber, whereas the subjects were exposed to LBNP 60 in the second and fourth sequences. We randomized the subjects to undergo CPT in the first and fourth or in the second and third sequences by pseudorandom numbers generated in Microsoft Excel 2010 (Microsoft). Between each sequence, the subjects returned to a pain‐free, comfortable situation with normal sense of temperature in the right hand. They could abort exposure to LBNP and CPT at request at any time. LBNP was relieved if they displayed symptoms or signs of impending circulatory collapse (gray out or drop in BP or HR). An intravenous cannula was not routinely placed, but an anesthesiologist with access to equipment for resuscitation was available during the experiments.

### 2.2 Measurements and data management

We measured ascending aortic blood velocity with a 2 MHz pulsed suprasternal Doppler probe (SD‐50; Vingmed Ultrasound) with a 3‐lead ECG. For continuous noninvasive arterial blood pressure (ABP) waveform we used the volume‐clamp method (Finometer; FMS Finapres Measurement Systems) around the left middle finger. We sampled the analog data at 400 Hz in custom‐made software (REGIST 3; Morten Eriksen, University of Oslo, Oslo, Norway) which were exported as .txt‐files to LabChart Pro v 8.1.18 (AD Instruments) for further analysis. We did also monitor the subjects with a pulse oximeter (Masimo Radical 7, software 7.3.1.1; Masimo) on the left index finger.

**FIGURE 2:**
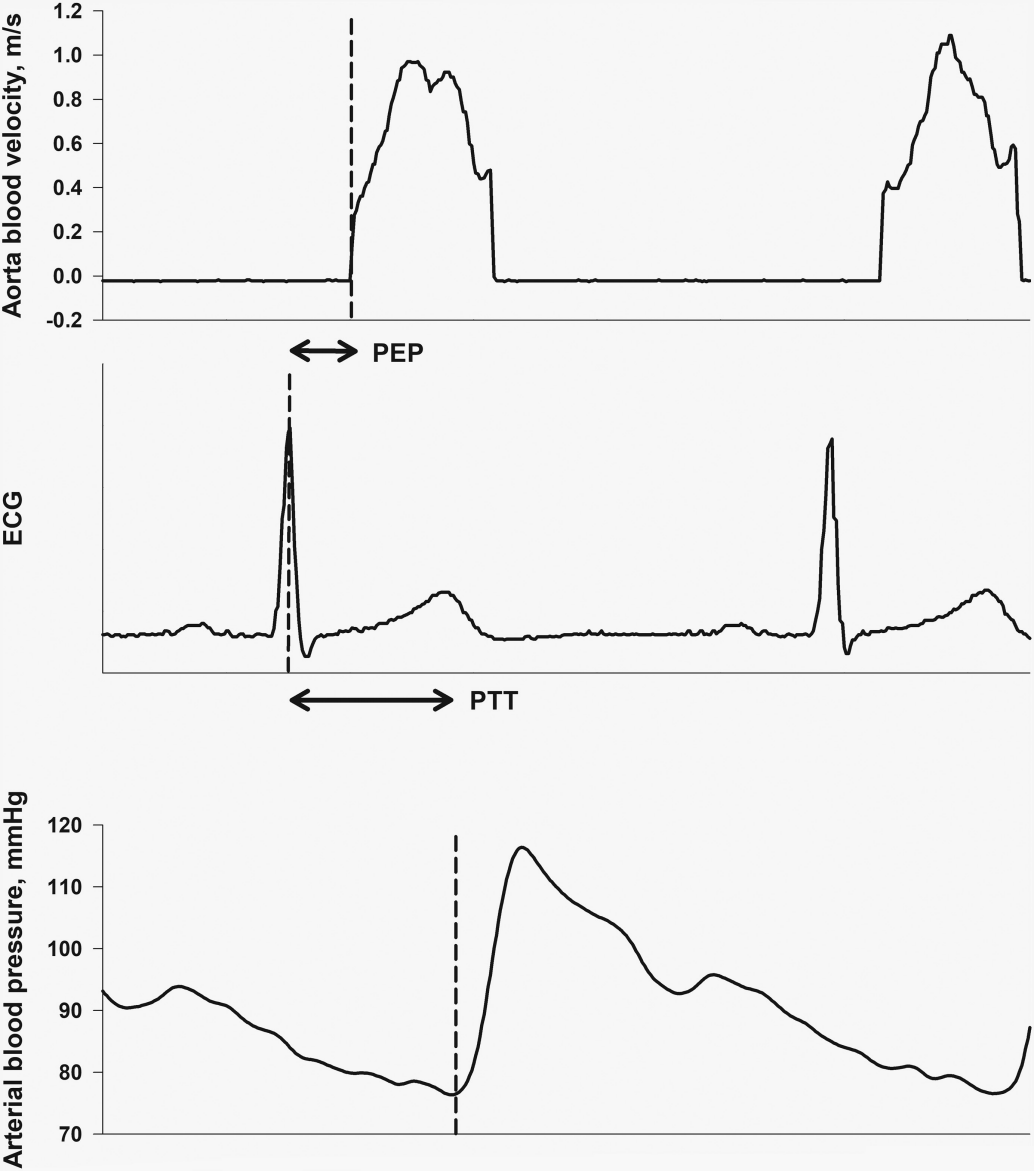
Pre‐ejection period (PEP) and pulse transit time (PTT).

We calculated pre‐ejection period as the time from the R spike of the ECG to the time when aortic flow velocity increased past a threshold of 0.1 m × s^−1^; a cutoff value to define the end of the isovolumetric contraction time (Figure 2). We smoothed the ABP waveform with a 100 ms triangular Bartlett window, and calculated pulse transit time from the ECG R‐peak to start upslope of the ABP waveform (Figure 2). We further calculated indexed pulse transit time as pulse transit time relative to the RR‐interval. In the supplementary material, we also present pulse transit time to maximal slope (1st derived) and pulse transit time to the maximal (peak) value of the ABP waveform (Figure 5, Appendix S1). Since there is not a uniform definition of pulse transit time in the literature, we chose to measure at all these different variants of pulse transit time.

We calculated HR from the RR‐intervals, while MAP and velocity‐time integrals (VTI) of aortic velocity were calculated as means and integrals within each RR‐interval of the ABP and aortic blood velocity waveforms, respectively. We calculated stroke volume from the aortic VTI, assuming a diameter of 20 mm and an angle of insonation of 20°. We calculated SVR as MAP × cardiac output^−1^. For further data handling, we used R 4.0.5 (R Core Team [2021]. R: A language and environment for statistical computing. R Foundation for Statistical Computing; https://www.R‐project.org) using RStudio 1.4.1106 (RStudio Team [2020]. RStudio: Integrated Development for R. RStudio, PBC; http://www.rstudio.com).

To explore physiological mechanisms behind the results, vascular transit time was calculated as the difference between pulse transit time and pre‐ejection period for each 30 s segment. Pre‐ejection period, vascular transit time, and pulse transit time for observations through the sequences were plotted against stroke volume, MAP, and systemic vascular resistance (SVR).

### 2.3 Statistical analysis

We calculated median values of pre‐ejection period, pulse transit time, and background hemodynamic variables for the 1‐min baseline and every 30 s thereafter, giving 14 values after baseline for each subject. Changes from baseline over time *within sequences* were calculated by assigning factors to each 30s segment (dummy variables) in linear mixed regression models with subjects as random effect (random intercept). Confidence intervals were adjusted for multiple measurements by the single‐step method of the glht‐function in the multcomp package in R (Hothorn et al., 2008). Differences *between sequences* were calculated from the last 30s segment with interventions (Figure 1) by assigning factors (dummy variables) in linear mixed regression models using the multcomp‐package, as above. By using qq‐plots, histograms and plotting residuals versus fitted values, we checked normality assumptions. We estimated associations in bivariate analyses as conditional R^2^‐values from linear mixed regression models (Nakagawa & Schielzeth, 2013) with observations clustered with subjects. We considered *p*‐values <0.05 statistically significant. Results are mean (SD) or median (25th, 75th percentiles) unless otherwise stated. We did not perform a separate power calculation for the present analysis.

## 3 RESULTS

**FIGURE 3:**
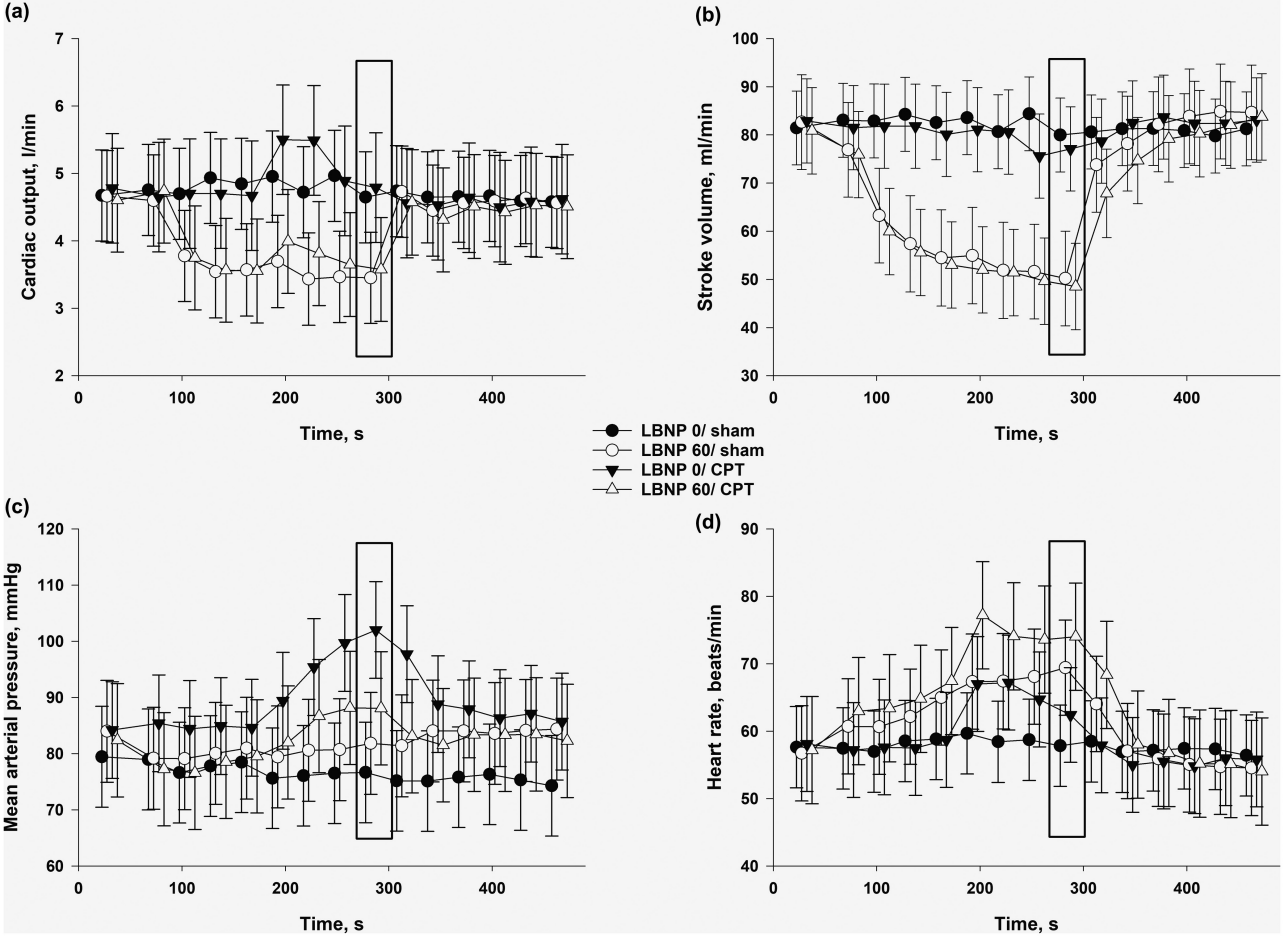
Changes from baseline for the different sequences for (a) cardiac output, (b) stroke volume, (c) mean arterial pressure, and (d) heart rate. The bar indicates the point where we have done our analyses, which represent the last 30 s of interventions.

Twenty subjects (10 men) were included in the original study. We experienced noise on the ECG‐signal that made it unsuitable for the current analyses in six of the subjects, leaving 14 subjects available for the current analyses with age 26 years (21, 24), weight 73 kg (66, 80), and height 179 cm (176, 180). One subject experienced symptoms of impending circulatory collapse during the CPT/LBNP 60‐sequence; all other subjects completed all sequences. In Figure 3, we present MAP, HR, stroke volume, and cardiac output through the sequences.

### 3.1 Pre‐ejection period

**FIGURE 4:**
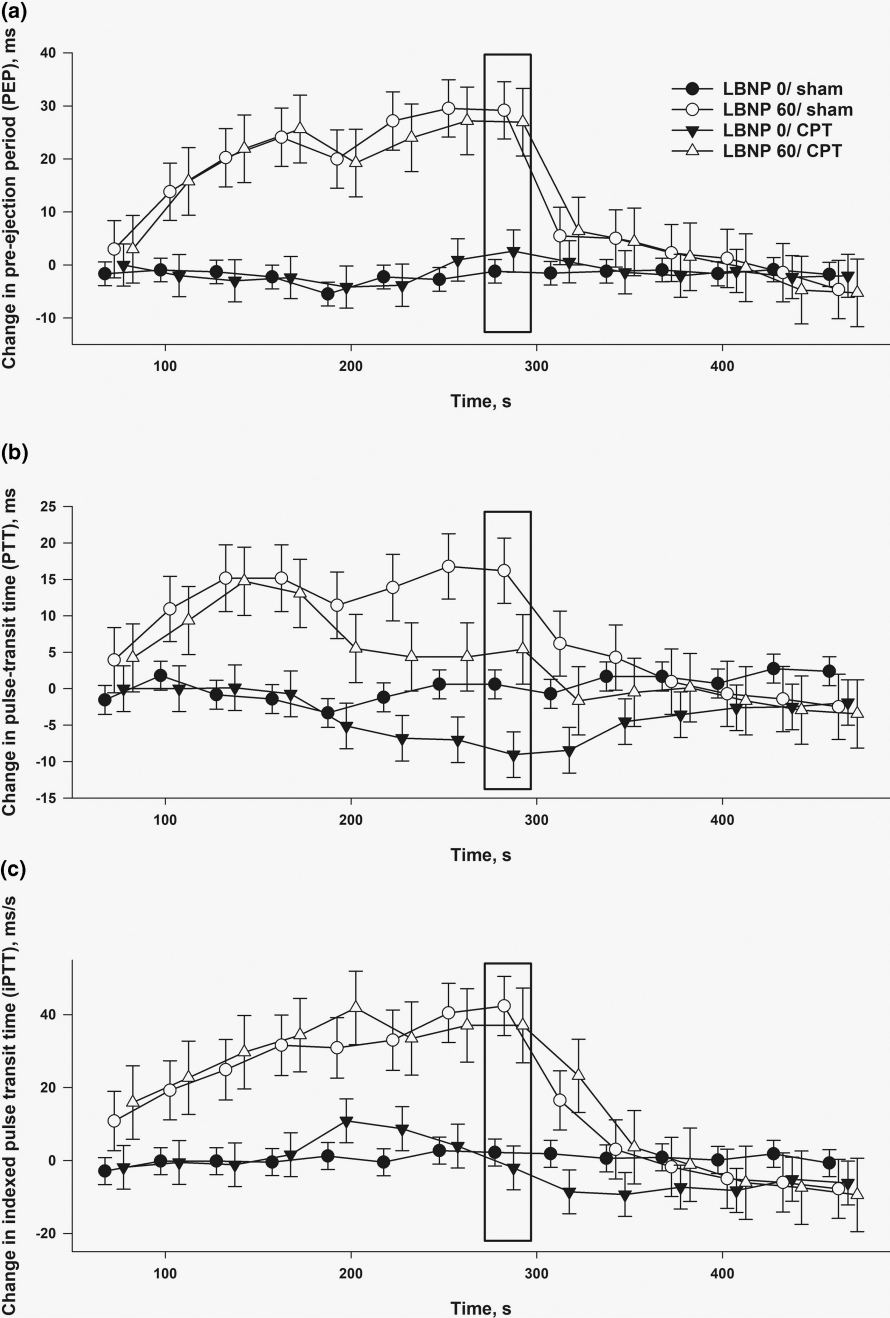
Changes from baseline for the different sequences for (a) pre‐ejection period (PEP), (b) pulse transit time (PTT), and (c) indexed pulse transit time (iPTT). The bar indicates the point where we have done our analyses, which represent the last 30 s of interventions.

Compared to baseline, pre‐ejection period increased *within sequences* over time with LBNP 60, both with and without CPT, but did not change with CPT alone (LBNP 0/CPT) (Figure 4, upper panel; Table 1). Comparisons *between sequences* showed the same pattern of significant differences between the sequences with and without LBNP 60 (Table 2).

**TABLE 1:** Estimates with 95% CI and p‐values for changes of pre‐ejection period (PEP), pulse transit time (PTT), and indexed pulse transit time (iPTT)

	PEP, ms	PTT, ms	iPTT, ms/s
LBNP 0/sham	−1.2 (−9.0 to 6.6, *p* = 0.99)	0.5 (−5.5 to 6.7, *p* = 1.0)	2.2 (−9.3 to 14, *p* = 0.98)
LBNP 60/sham	29 (21 to 37, *p* < 0.001)	16.2 (10 to 22, *p* < 0.001)	42 (31 to 54, *p* < 0.001)
LBNP 0/CPT	2.6 (−5.2 to 10, *p* = 0.88)	−9.0 (−15 to −2.9, *p* = 0.001)	−2.0 (−14 to 9.5, *p* = 0.99)
LBNP 60/CPT	27 (19 to 35, *p* < 0.001)	5.7 (0.0 to 12, *p* = 0.11)	38 (26 to 49, *p* < 0.001)

*Note*: Changes are from baseline to last 30 s of interventions under each experimental condition.

**TABLE 2:** Estimates with 95% CI and p‐values for the difference between sequences for (a) pre‐ejection period (PEP), (b) pulse transit time (PTT), and (c) indexed pulse transit time (iPTT)

	LBNP 60/sham, ms	LBNP 0/CPT, ms	LBNP 60/CPT, ms
(a) PEP			
LBNP 0/sham, ms	30 (19 to 42, *p* < 0.001)	3.8 (−7.6 to 15, *p* = 0.83)	28 (16 to 40, *p* < 0.001)
LBNP 60/sham, ms		−27 (−38 to −15, *p* < 0.001)	−2.2 (−13 to 9.0, *p* = 0.96)
LBNP 0/CPT, ms			24 (13 to 36, *p* < 0.001)
(b) PTT			
LBNP 0/sham, ms	16 (6.7 to 25, *p* < 0.001)	−9.6 (−19 to −0.7, *p* = 0.03)	5.1 (−4.1 to 14, *p* = 0.48)
LBNP 60/sham, ms		−25 (−34 to −16, *p* < 0.001)	−10 (−20 to −12, *p* = 0.02)
LBNP 0/CPT, ms			15 (5.6 to 24, *p* < 0.001)
(c) iPTT			
LBNP 0/sham, ms	40 (23 to 57, *p* < 0.001)	−4.2 (−21 to 13, *p* = 0.92)	35 (18 to 52, *p* < 0.001)
LBNP 60/sham, ms		−44 (−61 to −28, *p* < 0.001)	−4.9 (−22 to 12, *p* = 0.88)
LBNP 0/CPT, ms			40 (23 to 56, *p* < 0.001)

### 3.2 Pulse transit time

Compared to baseline, pulse transit time decreased *within sequences* over time with CPT without LBNP (LBNP 0/CPT) and increased with LBNP 60 without CPT (LBNP 60/sham) (Figure 4, middle panel; Table 1). For comparisons *between sequences*, we correspondingly found a lower pulse transit time with LBNP 0/CPT versus LBNP 60/sham, and both these sequences were different from and on either side of LBNP0/sham. We found no difference between LBNP 0/sham and LBNP 60/CPT (Table 2). Indexed pulse transit time increased with LBNP 60 with and without CPT but did not change with CPT alone (Figure 4, lower panel; Tables 1 and 2). Pulse transit time to maximal slope (1st derived) and pulse transit time to the maximal (peak) value of the ABP waveform showed the same pattern as pulse transit time to start upslope of the ABP waveform, and are presented in the Appendix S1 (Figure 6).

In the bivariate analyses of pre‐ejection period, vascular transit time and pulse transit time versus stroke volume, MAP, and SVR, the strongest linear relationship was between increases in pre‐ejection period and decreased stroke volume (Figure S7), whereas vascular transit time decreased with increased SVR (Figure S15).

## 4 DISCUSSION

The main findings in this study were that pre‐ejection period increased with hypovolemia, but did not change with pain. Pulse transit time changed in opposite directions with hypovolemia and pain; pain was associated with a reduction and hypovolemia with an increase. When simultaneously inducing hypovolemia and pain, the resultant pulse transit time was not significantly different from that of normovolemia and sham.

### 4.1 Pre‐ejection period; response to hypovolemia and pain

Pre‐ejection period has been shown to increase with decreased preload due to the Frank–Starling mechanism (Newlin & Levenson, 1979). This fits well with our data that show prolonged pre‐ejection period during hypovolemia. Ahmed et al. (1972) validated pre‐ejection period as a measure of myocardial contractility, and the reduced force of contraction with reduced preload may be the main explanation for the prolonged pre‐ejection period during hypovolemia in the present data. Another possible explanation is that the initial sympathoexcitatory response seen during hypovolemia may cause vasoconstriction and increased resistance to ejection. According to Harris et al. (1967), vasoconstriction and increased afterload prolong pre‐ejection period as it will take longer time for ventricular pressure to exceed aortic pressure. This effect may be counteracted by simultaneously increased contractility, which in itself will tend to decrease pre‐ejection period due to beta‐adrenergic‐mediated decrease in isovolumetric contraction time (Harris et al., 1967). This may explain our findings of an unaltered pre‐ejection period during CPT.

### 4.2 Pulse transit time; response to hypovolemia and pain

Arteriosclerosis is a process that changes the characteristics of the media layer, which leads to reduced arterial compliance. Increased arterial stiffness increases pulse wave velocity, described by the Bramwell–Hill equation (Bramwell & Hill, 1922), and is therefore a hallmark of arteriosclerosis (Vlachopoulos et al., 2015). Vasoconstriction can explain why we found shortened pulse transit time during CPT. We believe that pulse transit time shortened during pain due to a reduction in vascular transit time, although this was only calculated indirectly in our study as *vascular transit time = pulse transit time – pre‐ejection period*. Vettorello et al. (2016) has shown that pulse transit time, or comparable terms of the same entity, is able to detect progressive central volume loss induced by LBNP and that pulse transit time can identify hemorrhage in trauma patients before cardiovascular derangement occurs with acceptable sensitivity and specificity (Vettorello et al., 2013). Chan et al. (2007) found that pre‐ejection period and pulse transit time increased significantly during hypovolemia. Pulse transit time has also been shown to increase with sudden hypovolemia during hemodialysis (Ahlstrom et al., 2005). These findings fits well with those of the present study.

In our previous analysis from the present study (Høiseth et al., 2015), we found that both hypovolemia and pain, which both activate the sympathetic nervous system (Kregel et al., 1992; Schadt & Ludbrook, 1991; Victor et al., 1987), reduced noninvasive measures of peripheral perfusion (somatic oximetry and perfusion index), making the distinction between the two stimuli difficult. In the present analysis, both stimuli also influenced pulse transit time, which may thus seem to suffer from the same limitations. However, pulse transit time changed in opposite directions and may thereby have the potential to accompany other diagnostic methods and differentiate between the effect of hypovolemia and pain. The opposite change of pulse transit time during hypovolemia (longer) and pain (shorter) can explain why hypovolemia and pain combined (LBNP60/CPT) could not be differentiated from normovolemia and no pain (LBNP0/sham).

Vettorello et al. (2013, 2016) calculated a modified pulse transit time called *heart‐to‐arm time*, indexed to the RR‐interval (iHAT). They found that iHAT was longer during experimental hypovolemia in healthy volunteers and in hemorrhagic compared to nonhemorrhagic patients who were non‐anesthetized and without analgesic treatment. Our findings for indexed pulse transit time correspond with these findings, as it did not change with pain alone. Calculating pulse transit time relative to the RR‐interval may thus potentially add additional value as it may be less confounded by other sympathetic stimuli. It remains to be elucidated how it would respond to hypovolemia combined with other causes of increased heart rate than pain, such as anxiety and fear.

### 4.3 Bivariate analyses

Based on the R^2^‐values of the bivariate analyses presented in Appendix S1, the strongest associations were for pre‐ejection period and vascular transit time versus stroke volume and SVR. However, the physiological and mathematical coupling of the variables complicates exploration of causal relationships (Walsh & Lee, 1998). For example, to maintain MAP, SVR will increase as stroke volume and cardiac output is reduced, and any variable associated with stroke volume may therefore also be associated with SVR (inversely). The results are nonetheless compatible with an increase in pre‐ejection period as preload decreases, and a decrease in vascular transit time as afterload and vascular stiffness increases.

### 4.4 Methodological considerations

The lower body negative pressure model is considered a valid model to examine the physiological responses to central hypovolemia (Goswami et al., 2019). Circulatory parameters, such as HR, MAP, stroke volume, and cardiac output, show similar response to LBNP and bleeding (Goswami et al., 2019). The LBNP‐model will however not display responses to tissue damage which may otherwise be seen in trauma patients (Cooke et al., 2004), and is also of relatively short duration. The findings thus need to be validated in hypovolemic traumatic patients. Our study subjects were young and healthy and thus representing a patient population that accounts for a large proportion of patients with traumatic injuries (Moran et al., 2018). However, the findings also need to be validated in older patients and patients with comorbidities. Our study subjects probably did not have significant arteriosclerosis, which otherwise could have affected our findings considering its influence on pulse wave velocity.

We examined *changes* in the variables from baseline, rather than absolute values. This is because a minor delay in signals may be large relative to the changes in pre‐ejection period and pulse transit time induced by the interventions. Further, different monitoring devices can have differing and unknown time delays. The approach of analyzing changes only assumes that time delays are constant within subjects, and we did not find reasons to believe otherwise when analyzing the data. We calculated pulse transit time using the waveform of a volume‐clamp device. As this is based on a photoplethysmographic technique, a waveform from an ordinary pulse oximeter could probably give similar results. However, when performing analyses on the waveform from a pulse oximeter used in the present study, it was apparent that minute and unpredictable drifts in the waveform invalidated this signal for the present analyses. It would however be of value to perform validation studies with ordinary pulse oximeters/photoplethysmographs, as these are near ubiquitous in emergency and critical care.

Our findings of the three measures of pulse transit time; to the start of the upslope, maximal upslope, or end of upslope of the arterial pressure waveform showed the same pattern. These measures thus seem interchangeable.

We assumed an angle of 20° to the Doppler beam and a diameter of 20 mm in calculations of cardiac stroke volume. We do not expect errors in these approximations to affect our results significantly, as they were based on changes from baseline.

## 5 CONCLUSION

In conclusion, this experimental study found that pre‐ejection period increased with hypovolemia and remained unaltered with pain. Pulse transit time was reduced by pain and increased with hypovolemia. Thus, by the direction of change in pulse transit time, one can potentially distinguish hypovolemia and pain. However, we did not find any significant difference between normovolemia and no pain (LBNP 0/sham) and hypovolemia and pain (LBNP 60/CPT), which challenges the use of pulse transit time as a noninvasive parameter for diagnosing hypovolemia among patients in pain. This favors the use of the indexed pulse transit time, which increased with hypovolemia with and without pain, but did not change with pain alone. Clinical studies are needed to investigate the use of pulse transit time in different patient populations and in different clinical settings to explore its feasibility and possible contribution as a parameter for detecting and tracking hypovolemia in trauma patients.

## AUTHOR CONTRIBUTIONS

J. Hisdal, S.A. Landsverk, and L.Ø. Høiseth designed and conducted the experiments. H. Djupedal, S.A. Landsverk, and L.Ø. Høiseth designed the current analyses. H. Djupedal, T. Nøstdahl, and L.Ø. Høiseth performed the current analyses. H. Djupedal drafted the manuscript. All authors edited and revised the manuscript. All authors approved the final version of the manuscript.

## ETHICS STATEMENT

The data for the present analysis were sampled for a study which has previously been published, approved by the regional ethics commitee (REK sør‐øst C, 2012/790). The participants were included after written informed consent.

## Supporting information




Appendix S1
