# Supplementary material for: Effects of experimental hypovolemia and pain on pre‐ejection period and pulse transit time in healthy volunteers
Source: Physiol Rep. 2022 Jun 24;10(12):e15355. doi: 10.14814/phy2.15355 (PMC9226798; doi:10.14814/phy2.15355)

Supplementary appendix

**Fig 5** Measures of pulse transit time to maximal upslope (PTT_deriv_) and end of upslope (PTT_max)_ of the arterial pressure waveform.

_

_

**Fig 6** Changes in pulse transit time to maximal upslope (PTT_deriv_) and end of upslope (PTT_max_) of the arterial pressure waveform from baseline for the different sequences. The bar indicates the point at where we have done our analyses, which represent the last 30s of intervention





**Table 3** Estimates with 95% CI and P-values for changes of pulse transit time to maximal upslope (PTT_deriv_) and end of upslope (PTT_max_) of the arterial pressure waveform. Changes are from baseline to last 30s of interventions under each experimental condition.

|  | **PTT_deriv_**, **ms** | **PTT_max_**, **ms** |  |  |
| --- | --- | --- | --- | --- |
| LBNP 0/ sham | -1.2 (-8.2 to 5.9,  P = 0.99) | 0.5 (-9.3 to 10,  P = 1.0) |  |  |
| LBNP 60/ sham | 20 (13 to 28,  P < 0.001) | 20 (10 to 30,  P < 0.001) |  |  |
| LBNP 0/ CPT | -11 (-18 to -3.5,  P = 0.001) | -13 (-23 to -3.1,  P = 0.004) |  |  |
| LBNP 60/ CPT | 9.6 (2.3 to 17,  P = 0.004) | 9.2 (-1.0 to 19,  P = 0.09) |  |  |

**Table 4** Difference between sequences. Estimates with 95% CI and P-values for changes of pulse transit time to maximal upslope (PTT_deriv_) and end of upslope (PTT_max_) of the arterial pressure waveform. Changes are from baseline to last 30s of interventions under each experimental condition.

| **PTT_deriv_** | LBNP 60/ sham, ms | LBNP 0/ CPT, ms | LBNP 60/ CPT, ms |
| --- | --- | --- | --- |
| LBNP 0/ sham, ms | 20 (5.2 to 34,  P = 0.002) | -13 (-28 to 0.9,  P = 0.076) | 8.8 (-5.9 to 23,  P = 0.41) |
| LBNP 60/ sham, ms |  | -33 (-47 to -19,  P < 0.001) | -11 (-26 to 3.7,  P = 0.22) |
| LBNP 0/ CPT, ms |  |  | 22 (7.5 to 37,  P < 0.001) |
| **PTT_max_** | LBNP 60/ sham, ms | LBNP 0/ CPT, ms | LBNP 60/ CPT, ms |
| LBNP 0/ sham, ms | 20 (5.3 to 34,  P = 0.002) | -13 (-28 to 0.9,  P = 0.075) | 8.8 (-5.9 to 23,  P = 0.41) |
| LBNP 60/ sham, ms |  | -33 (-47 to -19,  P < 0.001) | -11 (-26 to 3.7,  P = 0.22) |
| LBNP 0/ CPT, ms |  |  | 22 (7.6 to 37,  P < 0.001) |

# Scatterplots and linear regression

Scatterplots of changes in pre-ejection period (PEP), pulse transit time (PTT) and vascular transit time (VTT) to start upslope of the arterial pressure waveform *vs.* stroke volume, mean arterial pressure (MAP) and systemic vascular resistance (SVR) are presented with different sequences designated by color. Regression coefficients and conditional R^2^ values are presented, calculated in linear mixed models (random intercept) with data nested within subjects.

## Pre-ejection period (PEP)

### Fig 7 PEP *vs.* stroke volume

| Variable | Estimate | 95% CI | P-value | R^2^ |
| --- | --- | --- | --- | --- |
| Stroke volume, ml | -0.71 | -0.75 to -0.68 | <0.001 | 0.79 |


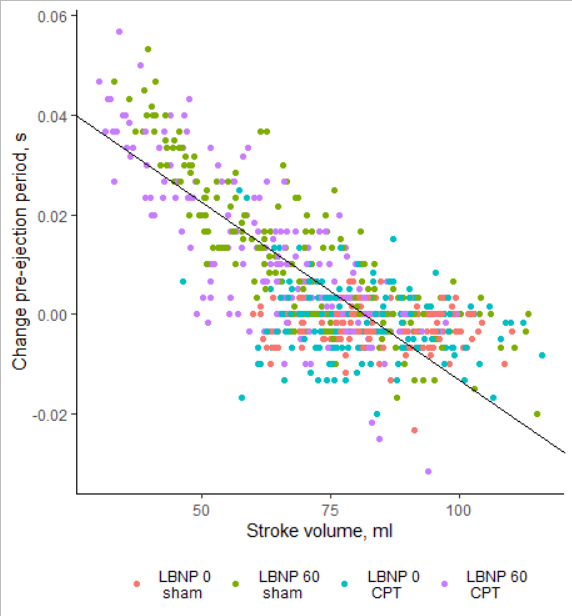


### Fig 8 PEP *vs.* MAP

| Variable | Estimate | 95% CI | P-value | R^2^ |
| --- | --- | --- | --- | --- |
| Stroke volume, ml | 0.036 | -0.040 to 0.11 | 0.36 | 0.05 |


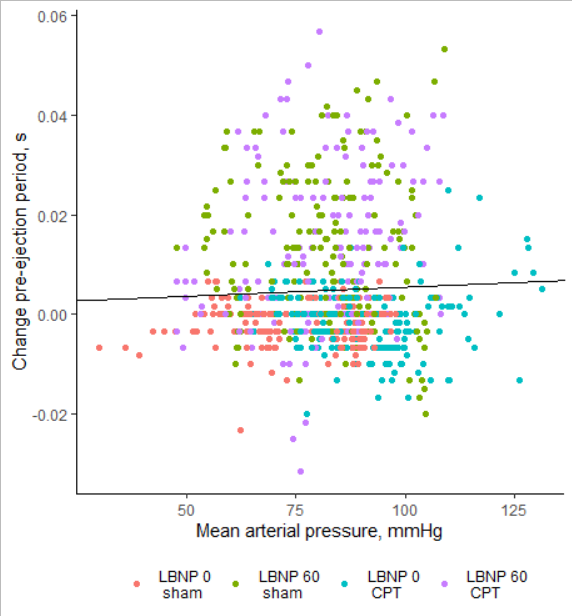


| Variable | Estimate | 95% CI | P-value | R^2^ |
| --- | --- | --- | --- | --- |
| Stroke volume, ml | 1.7 | 1.5 to 1.8 | <0.001 | 0.56 |

### Fig 9 PEP *vs.* SVR


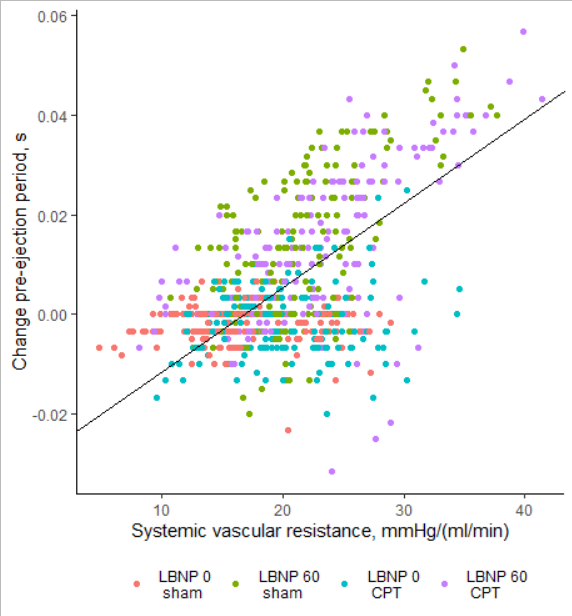


## Pulse transit time (PTT)

### Fig 10 PTT *vs.* stroke volume

| Variable | Estimate | 95% CI | P-value | R^2^ |
| --- | --- | --- | --- | --- |
| Stroke volume (ml) | -0.27 | -0.31 to -0.24 | <0.001 | 0.35 |
|  |  |  |  |  |


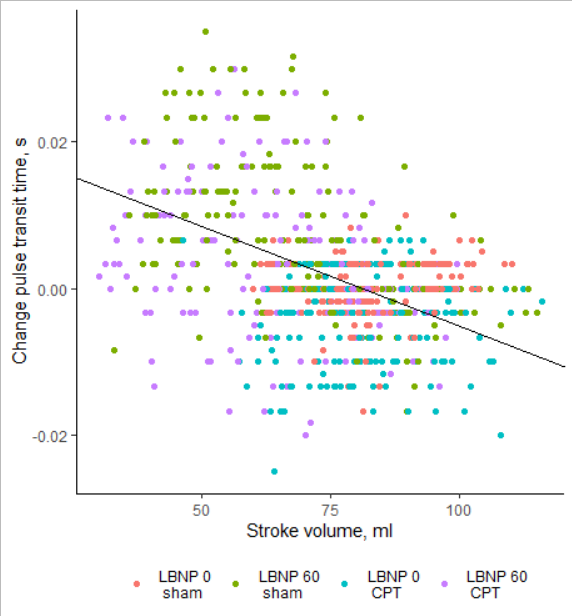


### Fig 11 PTT *vs.* MAP

| Variable | Estimate | 95% CI | P-value | R^2^ |
| --- | --- | --- | --- | --- |
| Stroke volume, ml | -0.3 | -0.35 to -0.24 | <0.001 | 0.35 |


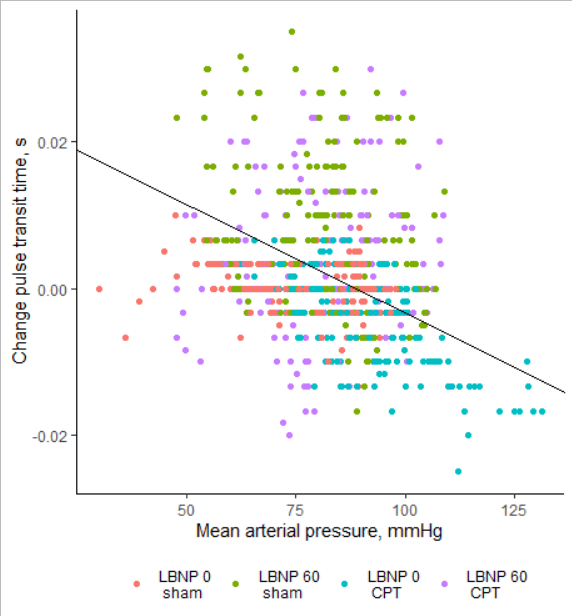


### Fig 12 PTT *vs.* SVR

| Variable | Estimate | 95% CI | P-value | R^2^ |
| --- | --- | --- | --- | --- |
| Stroke volume (ml) | 0.3 | 0.18 to 0.41 | <0.001 | 0.08 |


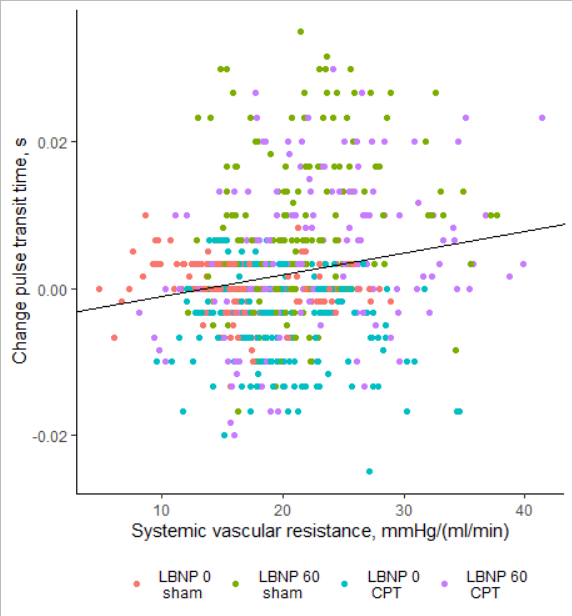


## Vascular transit time (VTT)

### Fig 13 VTT *vs.* stroke volume

| Variable | Estimate | 95% CI | P-value | R^2^ |
| --- | --- | --- | --- | --- |
| Stroke volume, ml | 0.43 | 0.40 to 0.47 | <0.001 | 0.54 |


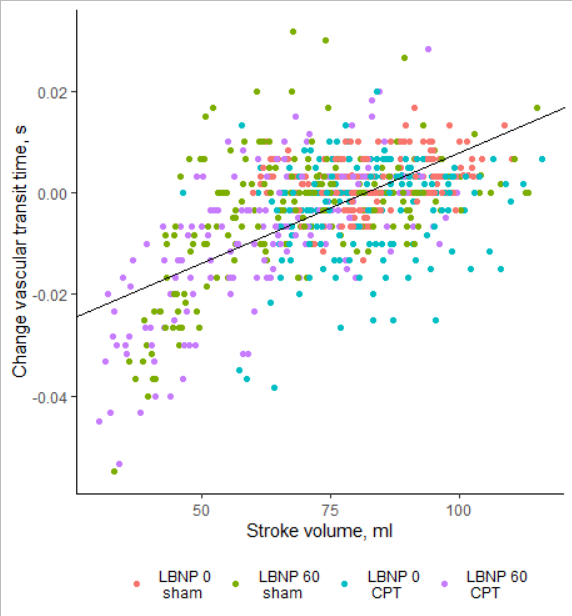


### Fig 14 VTT *vs.* MAP

| Variable | Estimate | 95% CI | P-value | R^2^ |
| --- | --- | --- | --- | --- |
| Stroke volume, ml | -0.36 | -0.42 to -0.29 | <0.001 | 0.40 |


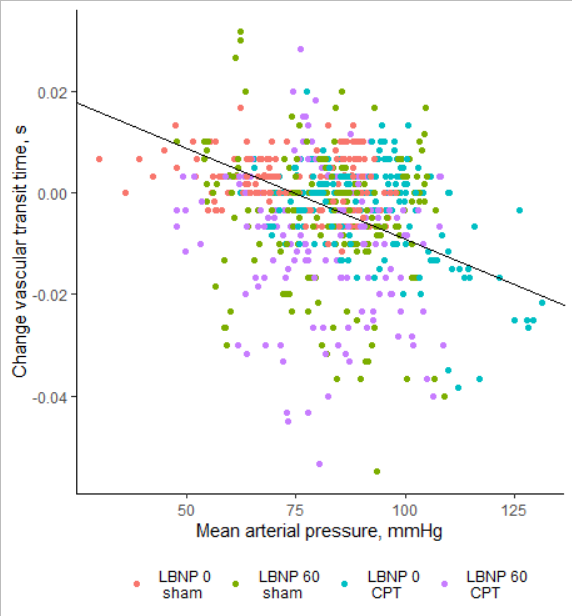


### Fig 15 VTT *vs.* SVR

| Variable | Estimate | 95% CI | P-value | R^2^ |
| --- | --- | --- | --- | --- |
| Stroke volume, ml | -1.4 | -1.5 to -1.3 | <0.001 | 0.61 |


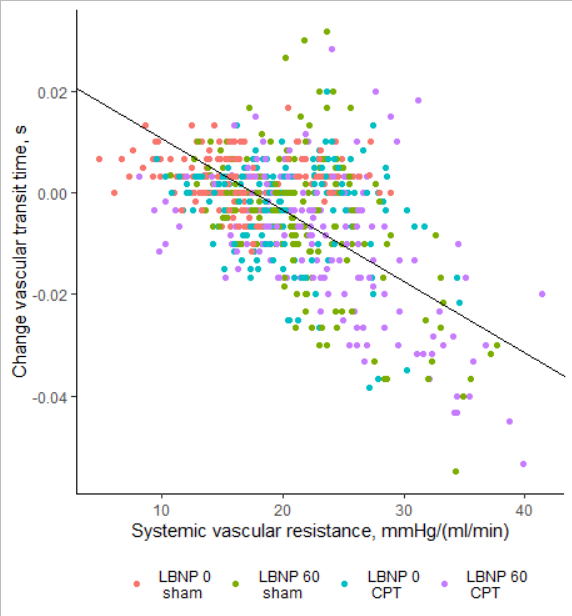

Supplement: Supplementary file 1 — Appendix S1 [file PHY2-10-e15355-s001.docx]
